# ChemTok: A New Rule Based Tokenizer for Chemical Named Entity Recognition

**DOI:** 10.1155/2016/4248026

**Published:** 2016-01-28

**Authors:** Abbas Akkasi, Ekrem Varoğlu, Nazife Dimililer

**Affiliations:** ^1^Computer Engineering Department, Eastern Mediterranean University, Famagusta, Northern Cyprus, Mersin 10, Turkey; ^2^Information Technology Department, Eastern Mediterranean University, Famagusta, Northern Cyprus, Mersin 10, Turkey

## Abstract

Named Entity Recognition (NER) from text constitutes the first step in many text mining applications. The most important preliminary step for NER systems using machine learning approaches is tokenization where raw text is segmented into tokens. This study proposes an enhanced rule based tokenizer, ChemTok, which utilizes rules extracted mainly from the train data set. The main novelty of ChemTok is the use of the extracted rules in order to merge the tokens split in the previous steps, thus producing longer and more discriminative tokens. ChemTok is compared to the tokenization methods utilized by ChemSpot and tmChem. Support Vector Machines and Conditional Random Fields are employed as the learning algorithms. The experimental results show that the classifiers trained on the output of ChemTok outperforms all classifiers trained on the output of the other two tokenizers in terms of classification performance, and the number of incorrectly segmented entities.

## 1. Introduction

Chemical domain is very active research field where there is a rapid accumulation of scientific articles and abstracts in repositories such as Medline and PubChem [1]. Therefore there is an imminent need for automatic extraction systems in order to mine useful knowledge from this vast amount of available information. Recently, there has been an increasing demand to facilitate various kinds of chemical information retrieval tasks from raw text, including scientific papers, books, patents, or technical reports. Named Entity Recognition (NER) is a subtask of information extraction (IE) that seeks to locate and classify elements of text into predefined categories such as names of locations, peoples, and organizations in newswire domain; protein; and gene names in the biomedical domain and types of compounds in the chemical/drug domain [2, 3]. It is usually regarded as a preliminary first step of many other text mining tasks such as relation extraction between chemical compounds, drug-drug relation detection, and event detection [4]. Therefore the success of the NER task may significantly affect the overall performance of these text mining applications.

Text mining in chemical domain has attracted significant interest from the text mining community in the recent years. Currently, one of the most important tracks of information extraction in chemical text mining tasks is to find valuable chemical/drug names in a given text. This task will be referred to as the ChemNER task in the discussion that follows. Due to the complexities of the language and the notation used in this domain, the success in ChemNER has yet to reach the levels achieved in other domains. ChemNER can be considered as a sequence labeling problem where the final goal is to assign a predefined class label to each part of the given text [4, 5]. Researchers in the field have utilized different types of NER systems such as rule based, dictionary based, and machine learning based as well as hybrid systems. However, most state-of-the-art systems are based on supervised machine learning algorithms which require text to be segmented into meaningful units known as tokens prior to subsequent processing [4]. Tokens can be defined as the smallest discrete units of meaning in a document relevant to the task at hand or the smallest entities of information that cannot be further reduced in form and still carry that information [6]. However, in the biomedical domain and more specifically in the chemical/drug domain, tokens do not necessarily carry meaning.

Various methods for tokenization of text exist in the biomedical/chemical domain. In order to extract noun phrases from Medline articles, Bennett et al. [7] used a white space tokenizer that was modified to accommodate embedded punctuation used extensively in the specialized nomenclature of the domain. Seki and Mostafa [8] used dictionary look up based approach for protein name extraction by using hand crafted rules and filtering to identify protein name candidates to check against their dictionary. At the tokenization step, Kayaalp et al. [9] normalized input data by converting all letters to lowercase, as well as grouping all consecutive white spaces into a single white space. Additionally, they looked for hyphenated variants of tokens containing both alphabetic and numeric characters. Barrett and Weber-Jahnke [10] considered tokenization as a classification task and proposed a method to build a biomedical tokenizer using the token lattice design pattern by adopting the Viterbi algorithm. Their classifier-based tokenizer splits or joins textual objects through classification to form tokens. Arens [6] introduced another classification based tokenization method for bioscience texts where the tokenization task is considered as a punctuation classification problem as delimiters and nondelimiters.

Two recent ChemNER systems that can be considered as state-of-the-art are tmChem developed by Leaman et al. [11] and Chemspot [12] is developed by Rocktäschel et al. [13]. tmChem is an open-source software tool used for identifying chemical names in biomedical literature. It uses Conditional Random Fields (CRFs) with a rich set of features and postprocessing modules [11]. tmChem utilizes the tokenization method employed in tmVar [14] which was used to extract sequence variants in biomedical text. This tokenizer is referred to as the tmVar tokenizer in the following discussion. tmVar tokenizer segments text into tokens at whitespaces, punctuations, digits, lowercase letters, and uppercase letters as well as special characters. Additionally, it splits strings on subsequent use of uppercase and lowercase letters. One of the most successful chemical NER systems, Chemspot, employs a hybrid method combining a CRF and a dictionary. Even though the tokenizer of Chemspot, henceforth referred to as Chemspot, is freely available for use, the implementation details are not provided.

It can be argued that, in general the shortcomings of a tokenization method may result in incorrect segmentation of named entities. However, to the best of the authors' knowledge there is no work which establishes a set of standard and globally accepted rules for tokenizing biomedical/chemical text. The effect of tokenizers on chemical or biomedical NER performance has not been investigated either. Although He and Kayaalp [16] compared 13 tokenizers used on Medline, by observing the tokenizer outputs, as Habert et al. mention in [17] there is no standard method for evaluating the quality of tokenization.

In this study, a rule based tokenizer which uses manually extracted rules from the training data set of ChemDNER task of BioCreative IV (BioCreative) is proposed. The proposed method aims at generating longer tokens without incorporating irrelevant text which may violate the Named Entity (NE) boundaries. Two state-of-the-art supervised machine learning algorithms used for sequence labeling, namely, Support Vector Machines (SVM) [18] and Conditional Random Fields (CRFs) [19], have been employed as classification systems. The effects of different tokenization methods have been investigated from two perspectives. Firstly the effect of tokenization on NER performance in terms of *F*-score is studied. Secondly the violation of the Named Entity boundaries caused by tokenization is analyzed. The latter is referred to as “incorrectly segmented entities.” Two of the most comprehensive chemical/drug related data sets, namely, BioCreative IV Critical Assessment of Information Extraction in Biology (ChemDNER) corpus [20] and SemEval 2013 Drug Name Recognition corpus [21, 22], are used in the experiments. We compare the performance of the NER classifiers which utilize the proposed tokenizer, named as ChemTok, to the tokenizers of two state-of-the-art systems recently employed in ChemNER, namely, tmChem and ChemSpot. It is important to note that, in this study, the emphasis is on studying the effects of the tokenization phase on the performance of the ChemNER task rather than the absolute NER performance. ChemTok can then be implemented in chemical NER systems.

## 2. Methods

### 2.1. Tokenization

Tokenization methods may vary depending on the context and the aim of the task [5, 23, 24]. The simplest tokenization method is breaking text into white space separated segments. In the commonly used newswire domain every token is equivalent to a word or a special character such as a punctuation mark or a digit. In some other domains such as the biomedical or chemical/drug domain, segmentation of text merely by using spaces would be neither adequate nor appropriate due to the variety of the nomenclatures utilized in the domain, inconsistent use of spaces, presence of punctuation marks inside NEs, constant addition of new terms, use of technical terminologies, nonstandard orthography, and existence of ambiguous punctuations [10, 25, 26].

For example, consider the following sentence from the article with PubMed ID 23403395 which contains four entities ***d-alpha-tocopheryl-co-pol***, (***TPGS*** *),* ***cisplatin***, and (***HER2***) (see [27]):

*We developed a nanocarrier system of herceptin-conjugated nanoparticles of** d-alpha-tocopheryl-co-poly**(ethylene glycol) 1000 succinate (**TPGS**)-**cisplatin** prodrug (HTCP NPs) for targeted co-delivery of cisplatin, docetaxel and herceptin for multimodality treatment of breast cancer of high human epidermal growth factor receptor 2 (**HER2**) overexpression.*

In the sample sentence given above, it can be seen that the first entity name d-alpha-tocopheryl-co-poly is conjoined with the nonentity text (ethylene glycol) and the two entity names TPGS and cisplatin are also combined into one token using dash (-) character. Note, on the other hand, that the dash character is integral to the entity (d-alpha-tocopheryl-co-poly). In both cases identifying the NEs is difficult due to the fact that tokenization does not match the original NE boundaries resulting in incorrectly segmented entities.

Consider the second sentence in article with PMCID 104802 which contains only one entity (see [28]):

*We describe a test which uses the ability of viable cells to reduce** 3-(4,5-dimethylthiazol-2-yl)-2,5-diphenyl tetrazolium bromide** (MTT) to detect resistance to a bactericidal drug, rifampin, in in vitro-cultured Mycobacterium tuberculosis.*

The entity ***3-(4,5-dimethylthiazol-2-yl)-2,5-diphenyl tetrazolium bromide***  contains white spaces inside the entity name itself. Using a white space tokenizer would result in the generation of 3 tokens:* “* ***3-(4,5-dimethylthiazol-2-yl)-2,5-diphenyl*** *”*,* “* ***tetrazolium*** *”, and “* ***bromide*** *”.* Furthermore, if the tokenizer segments text at punctuations the first token listed in the previous case would be broken down into a smaller tokens such as “3”, “-”, “(”, “4”. In order to accommodate the existence of multitoken NEs, NER tasks usually utilize the IOB tagging scheme [29] where the first token in a multitoken entity name is denoted by B-EntityName and all subsequent tokens are tagged as I-EntityName. Nonentity tokens are tagged as O. For example, the sentence fragment “inhibition of NF-kappa B activation” which contains an entity of type “chemical” is tokenized and IOB labeled as follows:

**Table:** 

inhibition	O
of	O
NF	B-CHEMICAL
-	I-CHEMICAL
Kappa	I-CHEMICAL
B	I-CHEMICAL
activation	O

Machine learning based approaches used for NER require tokenization followed by IOB labeling without prior knowledge of NE boundaries as preprocessing steps. As a result, incorrect segmentation of entity names during preprocessing may result in the failure of correct labeling of tokens during the classification stage which will have an adverse effect in the classification performance of the NER system.

### 2.2. Proposed Tokenizer

**Figure 1:**
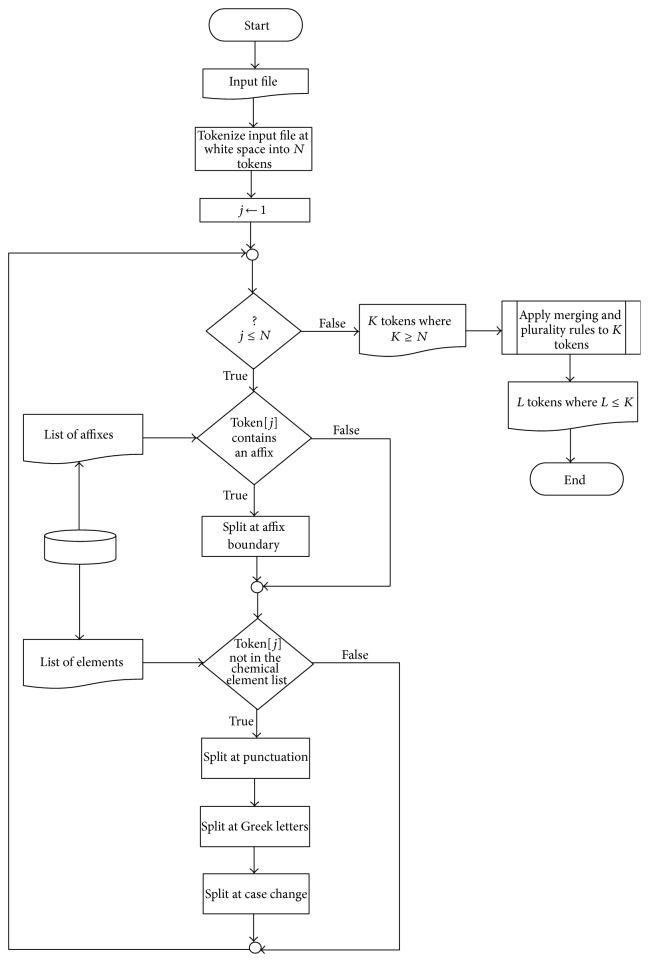
ChemTok Algorithm.

In this section, we propose ChemTok as a rule based tokenizer which uses manually extracted rules from the training data set of ChemDNER task of BioCreative IV. Overall, raw text is segmented at white spaces, numbers, all punctuation marks, and non-English characters such as Greek letters. Furthermore when two alphabetical characters with different cases are used subsequently without any space, the text is split into two tokens. Since learning algorithms try to find unique and distinctive patterns in order to categorize the tokens, it is important to produce samples which are as discriminant as possible. This goal can be achieved by generating the longest possible tokens without incorporating irrelevant text that may violate the NE boundaries. Consequently, the main novelty of the proposed tokenizer is the application of the extracted rules in order to merge the tokens split in the previous steps, thus producing longer and more discriminative tokens. The algorithm of ChemTok is depicted in Figure 1.

The first step of the algorithm simply tokenizes raw text at white spaces. The second step utilizes two lists. The first list contains domain specific affixes such as “Hyper”, “Anti”, and “Amino”, constructed from external sources listed in [15, 30]. The second list contains all chemical entities in ChemDNER train data. If a given token contains a substring that is found in the first list, then the token is segmented at the corresponding affix boundary.

For instance the tokens “Aminoacid”, “hyperinsulinaemia”, “Antiherpetic”, and so forth are split at this step. These conjoined tokens are separated into two tokens since these tokens can also be used separately as part of NEs. Following this step, the second list is used to decide whether a token will be considered for further tokenization or not. If the token is found in this list, it is assumed that the NE boundaries are correctly segmented and no further tokenization is required. The tokens that are not found in the second list are further tokenized at delimiters such as punctuation marks, Greek letters, and case changes of alphabetical characters.

The recombination rules (rules  1–4 from Table 1) in Step  3 are extracted from the ChemDNER train corpus in order to generate longer and thus more discriminative tokens. Rule  1 merges the tokens that were split incorrectly at punctuation marks. Rule  2 incorporates the balanced containers around digits into the token which is crucial for the recognition of formula entities in chemical domain.

Rule  3 is employed since Step  2.2 splits all words that start with uppercase, followed by sequence of lowercase including the common English words such as the ones which appear as the first word in a sentence. In Rule  4, the list of known chemical names containing chemical compounds, basic chemical elements, amino acid names, and amino acid chains [30, 31] is employed to merge tokens. A sliding window of 5 consecutive tokens is employed in a case insensitive search. Finally, Rule  5 strips the tokens in plural form into two tokens: one token for the base form of the word and one token for the plurality suffix such as “s”, “es” or “ies”. This case occurred frequently for entities in the SemeEval 2013 DDI corpus for both DrugBank and Medline training and test corpuses. Table 1 presents the rules used in Step  3 of the proposed algorithm and also gives an example for each rule.

## 3. Results and Discussion

In this section we compare ChemTok, with the tokenizers of two well-known ChemDNER systems, namely, tmVar and the tokenizer, for ChemSpot. The results are computed for two different data sets: ChemDNER data set [20] used in BioCreative IV event and the SemEval 2013 DDIExtraction Task 9.1 corpus [21].

BioCreative IV ChemDNER is an international community-wide effort that evaluates text mining and information extraction systems applied to the biomedical domain. ChemDNER task of BioCreative IV focuses on detection of mentions of chemical compounds and drugs, in particular those chemical entity mentions that can subsequently be linked to a chemical structure [4]. In particular, the entities are classified into one of the 7 chemical classes: abbreviation, formula, identifier, systematic, trivial, family, and multiple.

At present, the ChemDNER corpus is the most comprehensive publicly available chemical/drug related data set for NER task in the chemical domain. The corpus consists of 3 parts; training, development, and test data sets. The train and development sets contain 3500 abstracts each, and the test data set contains 3000 abstracts.  Table 2 shows the details of each data set. All sets include raw abstracts and annotation files listing each Named Entity together with its exact position in the corresponding abstract using character offsets.

The SemEval 2013 DDIExtraction task which also focuses on the biomedical literature consists of two challenges: (i) recognition and classification of drug names and (ii) extraction and classification of drug-drug interactions [21]. In this study only the corpus of first task, namely, Task 9.1, is considered. This corpus consists of 826 texts (626 DrugBank texts and 200 Medline abstracts). Each subcorpus is further split into two separate sets, as train and evaluation data sets. The corpus is distributed in XML format which contains character offsets of the NEs. The entities in this corpus belong to one of the 4 classes: drug, drug_n, brand, and group.  Table 3 presents details on the DDI corpus used in the study.

Table 4 shows the number and length of tokens produced by a generic white space tokenizer, ChemSpot tokenizer, tmVar, and ChemTok for each of the data sets described above. Additionally the number of incorrectly segmented entity names is given as the third column for each data set.

The phenomenon we refer to as the incorrectly segmented entities is important since the NER classifiers will not be able to identify NEs correctly if the entity names are not segmented at the boundaries. In addition to incorrect segmentation problems associated white space tokenization whose examples were given in Section 2.1 several other factors lead to incorrect segmentation even when other types of tokenizers are used. For example, often an entity name appears in its plural form in text, such as “*clonidines*” or “*salicylates*” where the actual entity is annotated as “*clonidine*” or “*salicylate*”. Such plural forms are usually incorrectly segmented by many tokenizers. Due to the use of Rule  5 in Step  3 of the proposed algorithm ChemTok does not suffer from plural forms. In some other cases the desired entity name appears as part of a longer text such as the case of “*hyperinsulinaemia*” where the annotators annotate “*insulin*” as the NE. Finally, sometimes NEs are wrongly joined to other parts of text during various stages of text preprocessing such as the example of “*CONCLUSIONGlucose*” where the annotators mark “*Glucose*” as the NE but a tokenizer which uses a rule to split NEs at the point where there is a case change will incorrectly segment the NE to “*lucose*”. The second and third type of incorrect segmentation is very difficult to detect.

It is depicted in Table 4 that the generic white space tokenizer tokenizes the text into fewer number of longer tokens but produces very large number of incorrectly segmented entities compared to all other tokenizers. On the other hand it can be observed that even though ChemTok produces slightly longer tokens compared to ChemSpot and tmVar, the number of incorrectly segmented entities is minimized showing that the boundaries of NEs are correctly identified by the proposed tokenizer. This effect is most evident on the train and development sets of the ChemDNER corpus as well as the train set of the DrugBank data set. Although, the decline in the number of incorrectly segmented entities when ChemTok is used on the ChemDNER corpus is expected, the fact that there is a pronounced decline in a new corpus, namely, DrugBank train data, suggests that the rules used generalize well to other corpora in this domain.

In order to further investigate the impact of the tokenization approaches discussed on NER performance, several classification experiments are conducted using data tokenized by each of the three tokenizers. Two state-of-the-art classification algorithms, SVM and CRFs, were used for this purpose. Yamcha [32] toolkit has been used for realizing the SVM, whereas Mallet [33] toolkit has been used for implementing the CRFs classifiers. Both toolkits are trained using default settings. In particular, the SVM employed by Yamcha is trained in the one-versus-all mode with a second-degree polynomial kernel. The cost per unit violation of the margin is set to 1 and the tolerance of the termination criterion is set to 0.001. Yamcha has been used in the forward parsing mode.

SimpleTagger interface of Mallet is used with default parameters where the number of iterations is set to 500 and Gaussian variance is 10. All systems were trained using the set of features which have been used successfully for NER in this domain.  Table 5 shows the list of features used grouped according to their types. The exact features used in each group are shown in column 2 of Table 5. Features from 7 different groups have been used resulting in 25 distinct features.

In order to conduct experiments on the BioCreative IV corpus, the train data for this corpus is used to train the classifiers. The recognition performance is tested on the development set and the test set separately. As previously mentioned, the SemEval 2013 DDI data consists of two corpora, namely, DrugBank and Medline. Each of these corpora consists of separate train and test data sets. The NER performance of the classifiers on the DDI data is studied using train and test data sets of DrugBank and Medline corpora separately. Standard *F*-score is used in order to compare the classifier performances.

The effect of tokenization on ChemNER is discussed from two different perspectives: the overall NER performance and class based performance.  Table 6 shows the microaveraged overall *F*-scores obtained when the two classifiers trained on BioCreative train data are tested on development and test data sets separately.

Similarly, Table 7 shows the microaveraged overall *F*-score obtained when the classifiers are trained and tested on DDI DrugBank and Medline corpora.

It can be seen from Tables 6 and 7 that the overall NER performance of the classifiers which used the white space tokenizer is very inferior compared to that of all other classifiers as a consequence of the large number of incorrectly segmented entities. On the other hand the performance of the classifiers utilizing ChemTok is higher regardless of the learning algorithm.

The higher improvement over the other two tokenizers when the BioCreative data is used to test the performance can be attributed to the fact that ChemTok uses rules extracted from the BioCreative data set. Nevertheless, it is evident in Table 7 that the NER systems using ChemTok still outperform the NER systems using the other two tokenizers indicating that the proposed rules generalize well to other data sets in the domain. It can also be observed from Table 7 that the classification performance achieved using DugBank data set is much higher compared to the performance achieved on Medline data set regardless of the learning algorithm and tokenizer used. In fact this result is not surprising since similar results have already been reported by all participating teams in the SemEval 2013 DDIExtraction Task 9.1 [22]. This result may be due to two reasons. Firstly, the train data set of the DrugBank data set is almost 4 times larger than the Medline train data set (see Table 4). As is well known, the size of train data set has a big effect on classification performance. The second reason lies in the different compositions of the two data sets. Although the percentage of the most popular entity is “Drug” in both data sets, the numbers of other entity types differ largely (see Table 3). In fact 12% of all entities in the DrugBank set belongs to “Brand” entity where only 1.5% of all entities belong to this type in the Medline data set. On the contrary, 23% of entities belong to the “drug_n” type in the Medline set where this number remains at 1% for the DrugBank data set [34]. It is further known that the classification of entities in the “Brand” category is easier to recognize since they are usually short, easy, and unique entity names; however the entities in the “drug_n” category are more difficult to recognize due to the variations and complexity constituting the NEs [22]. The classification performance according each entity type is discussed next.

The effect of the proposed tokenization method on ChemDNER is further examined by checking the NER performance of the classifiers individually for each class (entity type) present in each data set. Since the performance of the white space tokenizer has been shown to be inferior to all other methods, the class based analysis of results for this tokenizer is excluded from the remaining of the discussion. Tables 8 and 9 illustrate the class based performance of each classifier using the three tokenizers on the BioCreative and SemEval 2013 DDI data sets, respectively.

It can be seen from the results in Table 8 that NER performance is improved for all classes present in the data set when ChemTok is used. Results in Table 9 show that the classifiers which utilize data tokenized by ChemTok perform better in 22 out of 24 possible comparisons that can be made with the classifiers which use the other tokenizers. The only 2 exceptions arise when tmVar outperforms ChemTok for class “Brand” when SVM is utilized in the DrugBank data set and for class “Drug_n” when CRFs are utilized for Medline data set. In the former case the difference in classification performance between tmVar and ChemTok is only 1%. The latter case takes place where the overall classification performance of all classifiers is very poor. In 8 cases, a comparison cannot be made since all classifiers fail to recognize any entities correctly in the particular class regardless of the learning algorithm or the data set used. This result can be explained by the fact that only 1% (130 out of 15756) of the entities in the DrugBank data set belong to class “Drug_n” and 1.5% (42 out of 2746) of entities in the Medline data set belong to class “Brand” [34].

Evidently, the improvement in NER scores obtained for individual classes is reflected in the overall *F*-scores presented in Tables 6 and 7. The observation that the use of ChemTok results in a NER improvement for almost all classes using three different data sets suggests that individual rules used by ChemTok affect each entity class depending on the structure of tokens used in the respective class. For instance, Step  2.1 of the proposed tokenization algorithm correctly segments at affixes resulting in a positive effect on the entities belonging to the “Family” class in the BioCreative data set and entities in the “Group” class in the SemEval DDI corpus. Step  2.2 of the algorithm ensures that tokens which are already known are not further tokenized improving the tokenization performance of the mentioned entities in the “Trivial” class and all entities in the SemEval DDI corpus. Rule  1 used at Step  3 makes correct segmentation at entity boundaries for entities which belong to “Multiple,” “Systematic,” “Formula,” and “Identifer” classes since the NEs in these classes contain digits and punctuations. Rule  2 mainly affects the entities in the “Formula” and “Systematic” classes which extensively use parenthesis in the entity names. Rule  3 improves tokenization for the entities in the “Trivial” class of BioCreative data set in addition to all the entities in the SemEval DDI data set through a lookup table of the known names.

## 4. Conclusions

Nonstandard nomenclature used in chemical text makes the use of standard tokenization methods for the ChemNER task difficult. In this study, a new rule based tokenizer which derives many of its rules from the train data set of a recent well known ChemNER task has been proposed. The proposed method is compared to the tokenizer components of two state-of-the-art ChemNER systems in terms of number of incorrectly segmented entities and overall NER performance using two learning algorithms, CRFs and SVM. Results suggest that ChemTok outperforms the other two tokenization methods in both aspects for two different data sets used for evaluation. Future work may involve the use of a larger corpus in order to improve the performance as well as devise possible new rules.

## Figures and Tables

**Table 1:** Rules used in Step 3 of the algorithm.

Rule number	Rule explanation	Example	
		Tokens after Step 2	Merged token
1	Numeric tokens which are separated by “.” or “,” or “/” or “-” or “_” are integrated into a single token.	125 , 12 , 12	125,12,12

2	If concatenated tokens from Rule 1 are surrounded by balanced containers such as parentheses, braces, and brackets, both container tokens are conjoined into the token.	( 1-3 )	(1-3)

3	Single uppercase tokens which are followed by sequence of lowercase letters as the next token are recombined to a single token.	C ommon	Common

4	If the concatenation of consecutive tokens is found in the list of known chemical names, they are merged into one token.	Na CL	NaCL

5	Apply the plurality rule to the tokens	Acids	Acids

**Table 2:** Details of BioCreative data set.

Data set	Number of abstracts	Number of sentences	Number of NEs in each class							Total number of NEs
			Systematic	Abbreviation	Family	Formula	Identifier	Multiple	Trivial	
Train	3500	30418	6656	4538	4090	4448	672	202	8832	29438
Development	3500	30445	6816	4521	4223	4137	639	188	8970	29494
Test	3000	8655	5666	4059	3622	3443	513	199	7808	25310

**Table 3:** Details of DDI corpus.

Data set	Number of documents	Number of sentences	Number of NEs in each class				Total number of Named Entities
			Drug	Group	Brand	Drug_n	
Train							
DrugBank	572	5675	8197	3206	1423	103	12929
Medline	142	1301	1228	193	14	401	1836
Test							
DrugBank	54	145	180	65	53	5	303
Medline	58	520	171	90	6	115	382

**Table 4:** Comparison of number of tokens, average token length, and number of incorrectly segmented entities for various tokenizers.

Data set	ChemSpot			tmVar			ChemTok			White space Tokenizer		
	NT	ATL	NISE	NT	ATL	NISE	NT	ATL	NISE	NT	ATL	NISE
Chem DNER												
Train	907405	4.62	40	965056	4.35	11	899343	4.66	**6**	718244	5.84	9189
Development	901610	4.64	36	958475	4.36	11	893180	4.68	**3**	714287	5.85	9174
Test	779700	4.63	8	828001	4.36	**3**	772847	4.67	**3**	513630	5.85	7804
DrugBank												
Train	127435	5.06	50	135625	4.76	48	126753	5.09	**6**	107409	6.00	4623
Test	3189	5.12	1	3407	4.79	1	3174	5.14	**0**	2665	6.12	116
Medline												
Train	32625	4.77	2	34178	4.55	2	32259	4.82	**1**	27066	5.75	431
Test	12978	4.85	**0**	13673	4.61	**0**	12875	4.89	**0**	10839	5.11	96

NT: number of tokens, ATL: average token length, and NISE: number of incorrectly segmented entities.

**Table 5:** Features used for training classifiers.

Feature set	Actual features in the feature set	Number of features used in set
Space features	Has right space, has left space, and has both right and left space	3

Context words	One token before and one token after current token	2

n-gram affixes	n-gram affixes (prefixes + suffixes) for *n* = 1 : 4 for each token	8

Word shapes	Word shape (number of uppercase, lowercase letters, digits, punctuation, and Greeks), digital word shape (word shape in digital format), and summarized word shape (combination of two aforementioned features)	3

Orthographic features	All uppercase, has slash, has punctuation, has real number, starts with digit, starts with uppercase, has more than 2 uppercase letters	7

Token length	Number of characters in the token	1

Common chemical prefixes and suffixes	Contains chemical affixes from the list of chemical affixes in [15]	1

**Table 6:** NER performance (*F*-score in %) of classifiers using BioCreative data set.

Tokenizer	Classification algorithm			
	CRF		SVM	
	Development	Test	Development	Test
White space	75.39	75.44	75.65	75.67
ChemSpot	78.46	78.89	83.26	82.88
tmVar	76.15	76.50	82.29	82.27
ChemTok	**81.77**	**81.89**	**85.15**	**84.94**

**Table 7:** NER performance (*F*-score in %) of classifiers using DrugBank and Medline corpora of DDI SemEval data set.

Data set	Tokenizer	Classification algorithm	
		CRF	SVM
DrugBank	White space	77.89	82.85
	ChemSpot	87.16	89.10
	tmVar	84.74	90.34
	ChemTok	**88.65**	**91.79**

Medline	White space	51.51	42.41
	ChemSpot	62.72	67.48
	tmVar	62.04	67.50
	ChemTok	**64.88**	**68.51**

**Table 8:** Class based performance (*F*-score in %) for BioCreative corpus using various tokenizers.

Algorithm	Entity type	Development set			Test set		
		ChemSpot	tmVar	ChemTok	ChemSpot	tmVar	ChemTok
CRF	Abbreviation	68.14	66.58	**68.68**	67.20	65.42	**68.86**
	Family	69.22	67.59	**72.67**	71.94	70.60	**74.91**
	Formula	76.57	69.70	**80.12**	75.29	69.81	**79.37**
	Identifier	63.03	59.45	**74.60**	63.88	61.55	**74.28**
	Multiple	32.50	26.86	**41.35**	32.77	30.50	**35.12**
	Systematic	79.41	78.09	**82.85**	79.95	78.33	**83.11**
	Trivial	85.62	84.11	**88.36**	85.52	83.69	**87.66**

SVM	Abbreviation	72.59	72.12	**74.50**	72.42	71.65	**73.75**
	Family	69.82	69.69	**71.99**	71.81	71.57	**74.31**
	Formula	82.667	81.61	**84.68**	82.15	81.68	**85.28**
	Identifier	72.08	69.76	**72.52**	74.60	74.76	**77.18**
	Multiple	36.06	34.31	**39.26**	26.89	20.96	**30.46**
	Systematic	82.33	81.51	**84.73**	82.10	81.49	**84.44**
	Trivial	86.73	85.85	**88.84**	86.50	86.14	**88.51**

**Table 9:** Class based performance (*F*-score in %) for SemEval DDI data set; DrugBank, Medline.

Algorithm	Entity type	DrugBank			Medline		
		ChemSpot	tmVar	ChemTok	ChemSpot	tmVar	ChemTok
CRF	Group	76.33	72.86	**79.16**	62.41	59.25	**64.31**
	Drug_n	0.0	0.0	**0.0**	10.44	**13.63**	12.48
	Brand	86.31	80.85	**89.97**	0.0	0.0	0.0
	Drug	89.77	86.85	**91.32**	74.57	74.22	**76.15**

SVM	Group	83.82	83.58	**85.12**	46.28	44.06	**49.13**
	Drug_n	0.0	0.0	0.0	10.93	11.02	**11.67**
	Brand	92.15	**94.11**	93.45	0.0	0.0	0.0
	Drug	91.66	89.32	**93.52**	68.04	67.06	**71.71**
